# MicroRNAs, Long Non-Coding RNAs, and Circular RNAs in the Redox Control of Cell Senescence

**DOI:** 10.3390/antiox11030480

**Published:** 2022-02-28

**Authors:** Daniele Lettieri-Barbato, Katia Aquilano, Carolina Punziano, Giuseppina Minopoli, Raffaella Faraonio

**Affiliations:** 1 Department of Biology, University of Rome Tor Vergata, 00133 Rome, Italy; daniele.lettieri.barbato@uniroma2.it (D.L.-B.); katia.aquilano@uniroma2.it (K.A.); 2 IRCCS Santa Lucia, 00179 Rome, Italy; 3 Department of Molecular Medicine and Medical Biotechnologies, University of Naples Federico II, 80131 Naples, Italy; carolina.punziano@unina.it (C.P.); giuseppina.minopoli@unina.it (G.M.)

**Keywords:** cell senescence, oxidative stress, redox homeostasis, aging, non-coding RNAs, microRNAs, long non-coding RNAs, circular RNAs

## Abstract

Cell senescence is critical in diverse aspects of organism life. It is involved in tissue development and homeostasis, as well as in tumor suppression. Consequently, it is tightly integrated with basic physiological processes during life. On the other hand, senescence is gradually being considered as a major contributor of organismal aging and age-related diseases. Increased oxidative stress is one of the main risk factors for cellular damages, and thus a driver of senescence. In fact, there is an intimate link between cell senescence and response to different types of cellular stress. Oxidative stress occurs when the production of reactive oxygen species/reactive nitrogen species (ROS/RNS) is not adequately detoxified by the antioxidant defense systems. Non-coding RNAs are endogenous transcripts that govern gene regulatory networks, thus impacting both physiological and pathological events. Among these molecules, microRNAs, long non-coding RNAs, and more recently circular RNAs are considered crucial mediators of almost all cellular processes, including those implicated in oxidative stress responses. Here, we will describe recent data on the link between ROS/RNS-induced senescence and the current knowledge on the role of non-coding RNAs in the senescence program.

## 1. Introduction

It is now established that cell senescence plays an increasingly significant role than first envisaged in the physio-pathology of the organism with already recognized beneficial and detrimental effects [1]. First, during embryogenesis, senescence has an active role in the normal development of different tissues [2,3]. Second, senescence controls tissue repair and homeostasis [4]. Third, senescence-induced cell cycle arrest can suppress tumor growth [5,6,7,8] and activate the clearance of tumor cells [9]. On the other hand, the accumulation of senescent cells during life may foster the onset of aging [10,11] and age-related pathologies, such as neurodegenerative diseases [12], cardiovascular pathologies [13], metabolic disorders [14], and eventually promote cancer [15,16,17]. Therefore, senescence has been proposed as a possible target to treat aging-related diseases and delay the aging process [18,19,20].

Endogenous reactive oxygen species (ROS) and reactive nitrogen species (RNS) are generated due to the aerobic metabolism or produced by specific enzymes, as well as following extracellular stimuli (recently reviewed in [21,22,23]). At physiological levels, ROS/RNS can fulfill important proliferative cascades and function as signal molecules [21,22,24]. However, due to their reactivity/toxicity, elevated ROS/RNS amounts trigger the accumulation of oxidative damages in the biomolecules, which are intercepted by endogenous sensors engaging downstream pathways to evoke a premature senescence program [25,26,27]. Senescent cells implement ROS/RNS production, which can in turn, enforce the proliferative arrest with further inadequate responses/effects [25,28,29].

There are multiple levels of regulation in the signaling network(s) initiating the cellular senescence program and sustaining it once activated. Most of these are based on transcriptional and posttranscriptional, as well as epigenetic changes highlighting not only proteins, but also different types of RNAs, including non-coding RNAs (ncRNAs), as crucial components of cell senescence program [27,30,31,32,33]. Non-coding RNAs constitute the majority of endogenous transcripts in the cells and comprise numerous RNA species grouped in different classes, based on their different lengths and activities. A well-known class includes “housekeeping” ncRNAs, such as ribosomal, transfer, and small nuclear/nucleolar RNAs with prominent roles in general cell functions. Another class comprises “regulatory” ncRNAs that groups RNA molecules with specific functions linked to the regulation of gene expression [33,34]. Among regulatory ncRNAs, microRNAs (miRNAs), long non-coding RNAs (lncRNAs), and more recently circular RNAs (circRNAs) [35,36,37,38] have been shown to play important roles in the physiopathology of organism cell senescence. Furthermore, ncRNAs are highly responsive to specific stimuli, which are involved in different cellular responses, including those evoked by oxidative stress [37], such as cell senescence.

The aim of the present review is to summarize the recent studies on the impact of miRNAs, lncRNAs, and circRNAs, as well as their regulatory integrated networks in cell senescence with particular attention to their functions in oxidative stress-induced senescence.

## 2. Cell Senescence as an Active Biological Program

**Figure 1:**
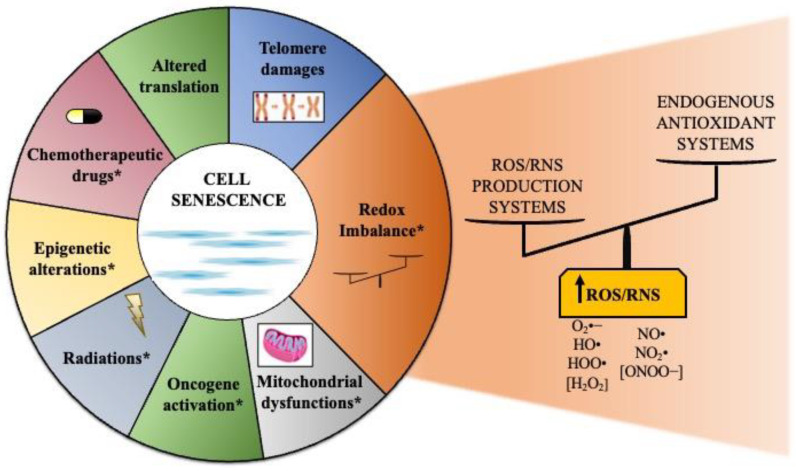
Intrinsic and extrinsic causes of cellular senescence. Schematic diagram showing the major inducers of cellular senescence. Damages to telomeres, redox imbalance, mitochondrial dysfunctions, activation of oncogenes, ionizing/ultraviolet radiations, epigenetic and chromatin alterations, chemotherapeutic drugs, and altered translation can promote the senescence program. Redox imbalance can be generated by decreased endogenous antioxidant defenses or increased ROS/RNS production. The main ROS/RNS are reported (the non-radical species are indicated in parenthesis). Most of these senescence inducers act by releasing ROS/RNS or by increasing ROS/RNS production. ROS/RNS can directly affect telomere integrity and cause altered translation process. * indicates senescence inducers that release or increase ROS/RNS.

Forty years ago, Dr. Leonard Hayflick provided the first evidence that primary human cells had a limited replicative capacity in culture (often referred to as the “Hayflick Limit”) [39]. The major determinant of this phenomenon, termed replicative senescence, is the length of telomeres [40]. Cells sense short telomeres as unresolved DNA damage and develop a double-strand DNA break response that irreversibly arrests proliferation [41,42]. However, telomere shortening is not the only cause of cell senescence. In fact, cell senescence is rapidly elicited in response to different types of stressors, such as increased oxidative stress [25,26,43,44], mitochondrial dysfunctions [45,46], aberrant oncogene activation [47,48,49], ionizing/ultraviolet radiations [50,51], epigenetic and chromatin perturbations [52], chemotherapeutic drugs [53], and altered translation process [54] (Figure 1). Of note, most of the senescence-inducing stimuli have been associated to ROS/RNS increase [28,55,56]. These intrinsic and/or extrinsic insults could directly or indirectly engage a DNA damage response (DDR) (reviewed in [42,57,58,59]). Therefore, cells halt proliferation and become prematurely senescent (premature or stress-induced senescence).

Despite their inability to replicate, senescent cells acquire specific features, such as morphological changes, metabolic and transcriptional reprogramming, as well as chromatin reorganization [27,60,61]. More importantly, they also express the so-called senescence-associated secretory phenotype (SASP) containing inflammatory chemokines and cytokines, growth factors, and proteases [62,63,64]. Of note, the SASP is cell type specific and can drive both physiological and pathological effects on local and/or distal tissues during life (reviewed in [20,27,59,65,66]).

Senescent cells are resistant to different apoptotic stimuli, mainly due to the increased expression of BCL-2 family proteins, such as BCL-W and BCL-XL [67]. Therefore, they are not eliminated, but rather accumulate in the organism, further sustaining redox imbalance, SASP and/or secretion of extracellular vesicles that also support non-autonomous activities [25,28,68]. Additionally, senescent cells exhibit resistance to ferroptosis, a non-apoptotic, iron-dependent form of cell death [69]. Increased iron in senescent cells could foster ROS/RNS increase and has been proposed as a new hallmark of senescence [25,28,70].

Even if senescence is a physiological response, widely recognized as an intrinsic tumor suppressor mechanism [48], a crucial contributor to embryonic morphogenesis [2,3] and to tissue repair [71,72,73], it is increasingly considered an important factor in aging and age-related pathologies (reviewed in [27,74]). Senescence has been observed both in mitotic and post-mitotic cells, as well as in adult stem cell pools of different tissues. Therefore, the accumulation of senescent cells impacts on the functionality of organs and limits also tissue renewal in vivo [27,74,75]. Importantly, senescence of mesenchymal stem cells (MSCs), now defined as mesenchymal stromal cells (MSCs), hinders their regenerative therapeutic potential [76].

## 3. Cell Senescence: A Focus on ROS/RNS-Mediated Pathways

The “oxidative stress theory of aging” postulates that cumulative oxidative damages to biomolecules, caused by “a disturbance in the prooxidant–antioxidant balance in favor of the former”, are responsible for the natural ageing process and that this can foster age-related pathological processes [77,78]. Despite the introduction of the concept of cell response to redox imbalance and recognizing a primary role of ROS/RNS in physio-pathological decline, this theory has gradually changed [79,80]. The next sections briefly discuss data linking oxidative stress to senescence.

### 3.1. Redox Homeostasis and Antioxidant Systems: Links to Senescence

Indeed, the term “Redox Homeostasis” encompasses a dynamic concept: Endogenous ROS/RNS, when produced in a regulated manner, are essential signaling molecules conferring outputs for living organisms, and thus their levels are finely regulated under physiological conditions [23,24,81]. On the one hand, this implicates that cells preserve physiological levels of ROS/RNS as normal oxidant cues (called eustress). On the other hand, cells neutralize ROS/RNS through intracellular defense systems to avoid oxidative challenges (termed distress) [21,82]. In fact, senescent cells fail to balance their intracellular redox status, skewing towards increased ROS/RNS that fuel senescence signaling and stabilization [25,28,29,83,84].

#### 3.1.1. ROS and RNS Generation and Cell Senescence

**Figure 2:**
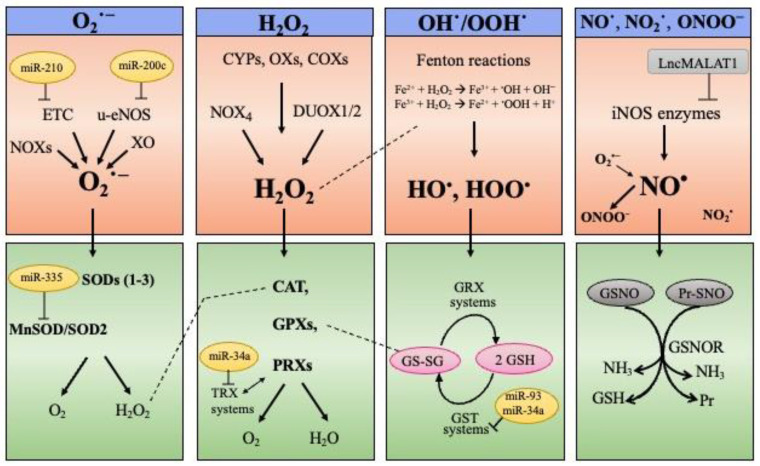
ROS and RNS biological sources and major scavenging pathways. ROS comprise the superoxide anion (O_2_^•−^), the hydroxyl (HO^•^), and hydroperoxyl (HOO^•^) radicals and the non-radical hydrogen peroxide (H_2_O_2_). Prevalent RNS are the nitric oxide (NO^•^), the radical nitrogen dioxide (NO_2_^•^), and the peroxynitrite anion (ONOO^−^). O_2_^•−^ can originate inside the mitochondrial electron transport chain (ETC) or from enzymatic reactions catalyzed by NAD(P)H oxidases (NOXs), xanthine oxidase (XO) or uncoupling nitric oxide synthase (u-eNOS). H_2_O_2_ is produced by NOX4, dual oxidase 1 and 2 (DUOX1/2), cytochrome P450s (CYPs), various oxidases (XOs), cyclooxygenases (COXs), as well as transiently by superoxide dismutase (SOD1–3) isoforms. HO^•^ and HOO^•^ are directly generated through the Fenton reactions. NO^•^ is produced by the enzymes nitric oxide synthases (NOSs), while the peroxynitrite anion (ONOO^−^) originates from the radical nitrogen dioxide (NO_2_^•^) combined with O_2_^•−^.

ROS and RNS are highly reactive molecules comprising free radicals and non-radical species (reviewed in [21,23,83,85]). ROS include the superoxide anion (O_2_^•−^), the hydroxyl (HO^•^), and the hydroperoxyl (H/ROO^•^) radicals, as well as the non-radical hydrogen peroxide (H_2_O_2_). They can originate as byproducts of the aerobic metabolism inside the mitochondrial electron transport chain (ETC) or from dedicated enzymatic reactions, as well as directly through the Fenton reactions (reviewed in [85,86,87]) (Figure 2). H_2_O_2_ represents the most relevant ROS: With a relatively low reactivity, it is the main component of signaling cascades and its principal targets are the thiol of cysteine residues, leading to changes in protein activities, as excellently reviewed elsewhere [21,88]. Moreover, H_2_O_2_ is regarded as the main source of HO^•^, which is the most reactive ROS provoking non-enzymatic oxidation in all of the biomolecules [21,89]. The prevalent RNS include the nitric oxide (NO^•^), produced by nitric oxide synthase (NOS) enzymes, the radical nitrogen dioxide (NO_2_^•^), and the highly genotoxic peroxynitrite anion (ONOO^−^) [23,85,86,89,90,91] (Figure 2).

The SOD1–3 are antioxidant isoenzymes involved in the dismutation of O_2_^•−^ producing H_2_O_2_, which is then inactivated by the enzymes catalase (CAT), glutathione peroxidases (GPXs), and peroxiredoxins (PRXs). GPXs use glutathione (GSH) to reduce substrates. Oxidized glutathione (GSSG) is reduced back by the glutaredoxin (GRX) system. PRXs instead form intramolecular disulfide bonds, which are then reduced via the thioredoxins (TRXs) system. GSH is also a cofactor of the GSH S-transferase enzymes (GSTs) that eliminate ROS/RNS along with xenobiotics and drugs. The enzyme S-nitrosoglutathione reductase (GSNOR) removes NO^•^ from S-nitrosoglutathione (GSNO) and nitrosylated proteins (Pr-SNO).

Some microRNAs and long non-coding RNAs (discussed in Section 4) affecting the stress/antioxidant networks are indicated.

Increased ROS/RNS levels can foster a cell switch into senescence [25,26,27,83,84]. Pioneer studies by Chen et al. first revealed that normal fibroblasts exposed to sub-lethal doses of H_2_O_2_ developed a senescence-like phenotype [43,44]. This phenomenon was later expanded to a plethora of conditions and/or external agents interrupting redox homeostasis [51,92,93,94,95]. For example, ROS generated by NAD(P)H oxidases, NOX1 and NOX4, are important players in premature senescence induced by oncogenic RAS [87] and constitutive active NOX4 mediates ROS-induced senescence in hematopoietic stem cells (HSCs) [96]. Similarly, increased NO^•^ levels or its derivatives has been described in pathophysiological processes as senescence/aging [90,91].

Excessive ROS/RNS derived from mitochondria were initially considered as a principal cause of cell senescence [28,92]. Mitochondria are the primary site of ROS/RNS production, but concomitantly they also represent the main targets of oxidative stress, as recently reviewed in [97]. Exposure to ROS/RNS can disrupt ETC integrity, which can in turn, feedback positively to enhance ROS/RNS levels, thereby sustaining senescence through a vicious cycle [98,99]. Moreover, healthy cells upon stress can replace damaged mitochondria through selective degradation (mitophagy) and biogenesis. On the contrary, during senescence, mitochondria increase both in number and size due to uncontrolled biogenesis [98,99,100] and/or to decreased Parkin-mediated mitophagy, involving ROS-mediated trapping of p53 in the cytosol [101]. Therefore, dysfunctional mitochondria contribute to impaired redox homeostasis and senescence.

#### 3.1.2. Antioxidant Systems and Cell Senescence

To cope with ROS/RNS toxicity, living cells use sophisticated endogenous antioxidant systems: Enzymatic and non-enzymatic scavengers, able to prevent (or quench) oxidative reactions, and consequently, cell physiology degeneration [21,102]. There is an enzymatic network to intercept and scavenge ROS/RNS (Figure 2). SOD1–3 isoenzymes located in different cell compartments are involved in the dismutation of O_2_^•−^ that produces H_2_O_2_ [103]. Zhang et al. observed increased oxidative damages associated with senescence markers in the kidney of SOD1-/- mice [104]. In addition, Kwon et al. reported premature-aging in transgenic mice with altered SOD3 function (R213G variant) [105]. H_2_O_2_ is inactivated by the enzymes catalase, glutathione peroxidases (GPXs), and peroxiredoxins (PRXs) (reviewed in [106]) (Figure 2). Catalase dysfunctions are associated with different oxidative stress-mediated diseases (reviewed in [107]) and cell models from GPX-/- mice featured senescence-like phenotype and increased susceptibility to H_2_O_2_ [108]. Additionally, PRX1-/- mice display reduced lifespan and fibroblasts lacking PRX1 are more susceptible to H_2_O_2_-induced damages [109]. GPXs use the tripeptide glutathione (GSH) to reduce substrates, while PRXs form intramolecular disulfide bonds, which are then reduced via the thioredoxins (TRXs) system [106] (Figure 2).

GSH behaves as a “guardian” of redox status, as well as a mediator of redox signal transduction [110] and some reports point to GSH depletion as the main culprits of oxidative stress favoring senescence [93,111,112]. Oxidized glutathione (GSSG) is reduced back to GSH by the glutaredoxin (GRX) system [113] (Figure 2). GRX1 has been recently linked to cell senescence. In fact, GRX1 silencing promotes ROS elevation and activates the p53 pathway [114]. GSH is also used by the GSH S-transferase enzymes (GSTs), that in addition to ROS/RNS, eliminate a wide range of toxic molecules, such as xenobiotics and drugs [115]. The antioxidant enzyme S-nitrosoglutathione reductase (GSNOR) removes NO^•^ from S-nitrosoglutathione (GSNO) and nitrosylated proteins, thereby modulating nitrosative stress signaling (reviewed in [116]) (Figure 2). Of note, GSNOR deficiency is associated with cell senescence and specific features of aging [117].

#### 3.1.3. NRF2 Antioxidant Pathways

An endogenous booster of redox homeostasis is the nuclear factor erythroid 2-related factor 2 (NRF2) [118,119,120,121,122]. Under unstressed conditions, newly synthesized NRF2 is degraded since the adaptor protein Keap1 (Kelch-like ECH-associated protein 1) ensures its continuous ubiquitination. Oxidative stress renders Keap1 inactive, thus NRF2 migrates into the nucleus where, mainly in the heterodimer with small musculoaponeurotic fibrosarcoma oncogene homolog (sMAF) proteins, orchestrate a selective transcriptional program aimed at restoring redox balance and counteracting possible deleterious effects due to ROS/RNS increase [120,121]. NRF2 reshapes antioxidant defense systems by inducing the expression of genes sustaining glutathione levels, such as those for GSH biosynthesis, GPXs, TRXs, and PRXs (reviewed in [118,119,120,121]). However, the NRF2-responsive genes are not limited to those implicated in thiol-based redox homeostasis since many others can function in detoxification, autophagy, and metabolism, as well as in anti-inflammatory cascades [118,119,120,121]. According to its cytoprotective role, NRF2 levels are reduced in senescent cells, as well as in aged tissues [123], thereby establishing persistent ROS/RNS production contributing to induction and maintenance of senescence (reviewed in [118]).

Mounting evidence also links non-coding RNAs with redox homeostasis, and thus with the control of senescence program. These molecules can modulate the redox status by targeting ROS/RNS producing or antioxidant enzymes (Figure 2). This aspect will be further discussed in Section 4.

### 3.2. ROS/RNS Signaling Pathways in Senescence (p53, p16, NF-κB, AMPK, SIRT, FOXO)

**Figure 3:**
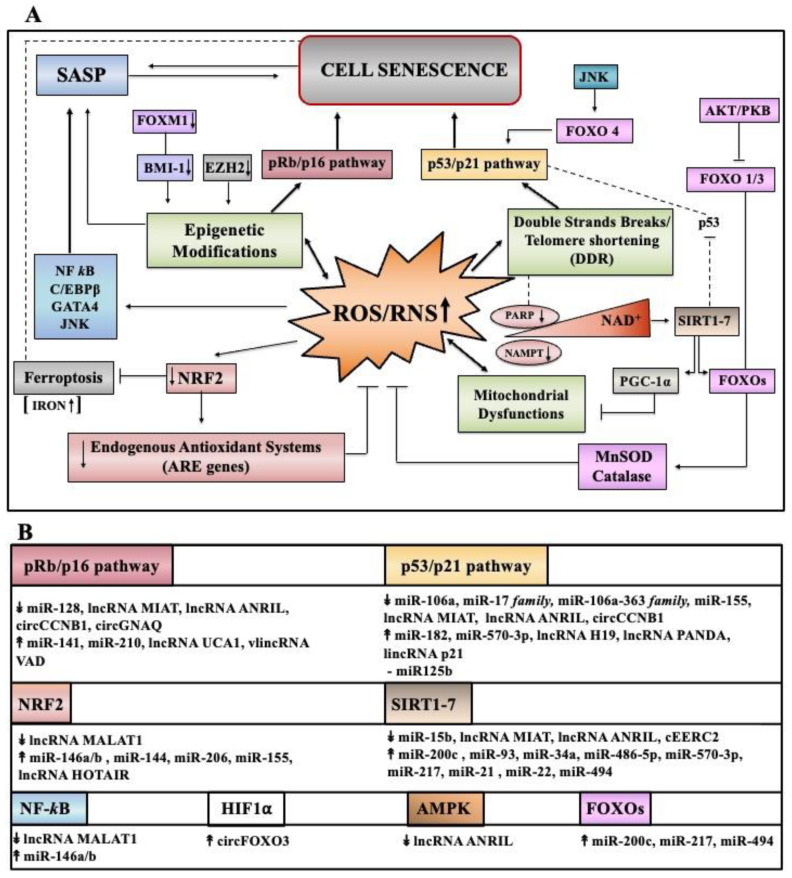
Summary of major pathways initiating senescence through ROS/RNS deregulation. (**A**) Failure of the antioxidant transcription program arising from NRF2 defects can induce senescence. In fact, NRF2 promotes the induction of numerous cytoprotective genes harboring ARE/s cis-element/s in their promoters. NRF2 also engages protective mechanisms to ferroptosis, thus preventing iron accumulation. Under redox imbalance, numerous molecular effectors/factors can mediate senescence-associated growth arrest. This relies on the activation of the p53/p21^CIP1^ and p16^INK4a^/Rb pathways. Persistent DDR activates the p53/p21^CIP1^ pathway, while epigenetic changes due to downregulation of BMI-1 and EZH2 proteins of the PCRs complex, induces p16^INK4a^/Rb pathway and establishes the SASP profile. SASP induction is mainly regulated by the redox-sensitive NF-κB pathway, along with C/EBPβ, GATA4, and JNK transcription factors. Dysfunctional mitochondria can also promote SASP (not shown). Mitochondrial dysfunctions increase ROS/RNS levels that also contribute to telomere damage and epigenetic modifications, and thus sustain senescence. Reduced levels of NAD^+^, a mitochondrial metabolite, affects the activities of sirtuins (SIRT1–7), thus provoking senescence by the p53/p21 pathway. Alteration of the NAD^+^/sirtuin pathway also impacts negatively on FOXO and PGC-1α activities, with consequent ROS elevation and mitochondrial dysfunctions. FOXO transcription factors increase catalase and SOD2 expression. FOXO1/3 are primarily inhibited by AKT and activation of AKT gives rise to senescence through increased intracellular ROS levels. FOXO4 activated by JNK induces senescence by engaging the p21 pathway. Low NAD^+^ amounts sustained by dysfunction of PARP1 and NAMPT enzymes also induce senescence. (**B**) Overview of non-coding RNAs deregulated in senescence that can modulate ROS/RNS signaling pathways. Up and down arrows indicate ncRNA levels that have been found as increased or decreased, respectively in various models of oxidative stress-induced senescence.

Loss of prooxidant–antioxidant balance, triggered by increased ROS/RNS production or inefficient antioxidant defense systems primarily due to NRF2 decline, provokes oxidative damages to biomolecules disturbing specific functions, signaling and/or metabolic pathways. This directly or indirectly (through a large set of molecular players) initiates and delivers signaling cascades leading to cell senescence (Figure 3A). In addition, ROS/RNS by inducing irreversible telomeric single-strand DNA breaks could foster the onset of senescence [55].

#### 3.2.1. Cell Cycle Arrest Pathways

DNA damage plays a critical role in ROS/RNS-induced premature senescence [27,43,55]. In particular, the double-strand DNA breaks initiate a DNA damage response (DDR), which if it becomes persistent converges on two DDR effectors: p53/p21 [7,48,49,50,62] and/or pRb/p16 [18,49,50] (Figure 3A). The oncosoppressor protein p53 arrests cell proliferation by inducing the expression of p21^Cip1^, a member of the cyclin-dependent kinase (CDK) inhibitor family. The pRb is a protein that in an hyphosphorylated state blocks proliferation by sequestering E2F/DP complexes necessary for cell cycle progression. The p16^INK4a^ is an inhibitor of other CDKs and, by preventing CDK-mediated phosphorylation of pRb, favors cell cycle arrest. The p21^Cip1^ also contributes to hyphosphorylated pRb since it can inhibit activities of other CDKs. However, activation of p53 and/or p21^Cip1^ tumor suppressor pathways can also occur in a DDR-independent manner [2,3]. DDR activation could also be a subsequent event of replicative stress caused by hyperproliferation stimuli initiated by NOX4-mitogenic ROS axis [124].

#### 3.2.2. SASP Pathways

In parallel to cell cycle arrest, the activation of redox-sensitive NF-κB signaling cascade drives the production of SASP (reviewed in [27,59,65]) (Figure 3A). Schreck et al. first showed that H_2_O_2_ mediates NF-κB activation [125]. NF-κB transcription factor is activated by various senescence-inducing pathways [62,126,127,128], and accordingly, inhibition of canonical NF-κB (RelA/p50) pathway delayed cellular senescence [129]. Among a vast repertoire of NF-κB-dependent genes, the inducible NOS (iNOS) can feed genotoxic peroxynitrite triggering ATM-mediated senescence [90]. There is also evidence recognizing a crosstalk between NF-κB and NRF2 in the senescence program [130]. Other transcription factors, such as CCAAT/enhancer-binding protein-β (C/EBPβ) and GATA4, as well as p38 MAP kinase and mammalian target of rapamycin (mTOR) signaling can intervene to produce selective secretome/s [20].

#### 3.2.3. Metabolic Pathways

As mentioned in Section 3.1.1, mitochondria homeostasis is essential for cell activities, and disturbances in their integrity/functions can induce senescence [99,100]. Mounting evidence suggests that beyond ROS/RNS increase, mitochondria can also trigger senescence through multiple cytosolic effectors/pathways [100]. First, bioenergetic imbalance can stimulate AMP-activated protein kinase (AMPK) that induces permanent arrest via increased p53/p21 in primary fibroblasts [131], and via pRb/p16 in endothelial cells [132]. Among mitochondrial metabolites, NAD^+^ levels when reduced can behave as a signal molecule in senescence and aging (reviewed in [133]) (Figure 3A). In line with this, nicotinamide phosphoribosyltransferase (NAMPT) enzyme involved in the NAD salvage pathway is downregulated during senescence and its re-expression delays senescence in MSCs [134].

Several studies have shown that if NAD^+^ declines, the activities of poly-ADP ribose polymerases (PARPs) and/or of deacetylase sirtuin (SIRT1–7) family are consequently inhibited and this can promote cell senescence (see References in [133]). Reduction of PARP1 activity can lead to activation of DDR signaling, in which PARP1 is a DNA damage sensor [133]. Furthermore, PARP1 regulates SIRT1 functions (directly or indirectly) since in addition to NAD^+^, they share various substrates (reviewed in [135]).

Beyond deacetylation of histones, SIRT1 deacetylates numerous transcription factors, including p53, PPARγ coactivator 1 alpha (PGC-1α), forkhead box O (FOXO), NF-κB, and hypoxia-inducible factor 1 (HIF-1α). By deacetylating p53, SIRT1 suppresses its transcriptional activity, and thus the low NAD^+^ can induce senescence sustained by the p53/p21 pathway [136]. SIRT3–5 enzymes are mainly located in the mitochondria and act preserving their homeostasis [137]. Reduced NAD^+^ negatively impacts the SIRT3/PGC-1α-dependent pathway [138], affects nucleus-mitochondria communication [139], and deregulates FOXO protective signaling along with mitochondrial unfolded protein response (UPRmt) [140,141] (Figure 3A).

#### 3.2.4. FOXO Pathways

FOXO transcription factors are involved in a plethora of biological functions, and thus they can affect senescence [141,142,143,144]. NAD^+^/sirtuin pathway post-translationally regulates FOXO activities that at least in many long-lived mutants, are causally involved in lifespan by modulating retrograde ROS signaling [145]. Since FOXOs increase catalase and SOD2 expression and FOXO1/3 factors are primarily inhibited by AKT/PKB (protein kinase B), the activation of AKT gives rise to cell senescence by increasing ROS [146,147,148]. The AKT role in oxidative stress-induced senescence was also proven in AKT-/- cells that display resistance to senescence [147]. On the contrary, FOXO4 fosters senescence via p21 increase, and is stimulated through the c-Jun N-terminal kinase (JNK) signaling [149]. Of note, a very recent article highlights a mitochondrial ROS-JNK signaling pathway to produce cytoplasmic chromatin fragments that trigger SASP [150]. Another member of the FOX family, FOXM1, is involved in the regulation of B cell-specific Moloney murine leukemia virus integration site 1 (BMI-1) expression and can counteract oxidative stress-induced senescence [151] (Figure 3A).

Finally, there is mounting evidence that the deregulated expression of numerous non-coding RNAs, especially microRNAs, can be causally involved in oxidative stress-induced senescence. As summarized in Figure 3B, they can impact the signaling cascades discussed above, via direct or indirect interactions with specific members of redox pathways, which will be covered in Section 4.

### 3.3. ROS/RNS: Links to Epigenetic Changes in Senescence

**Figure 4:**
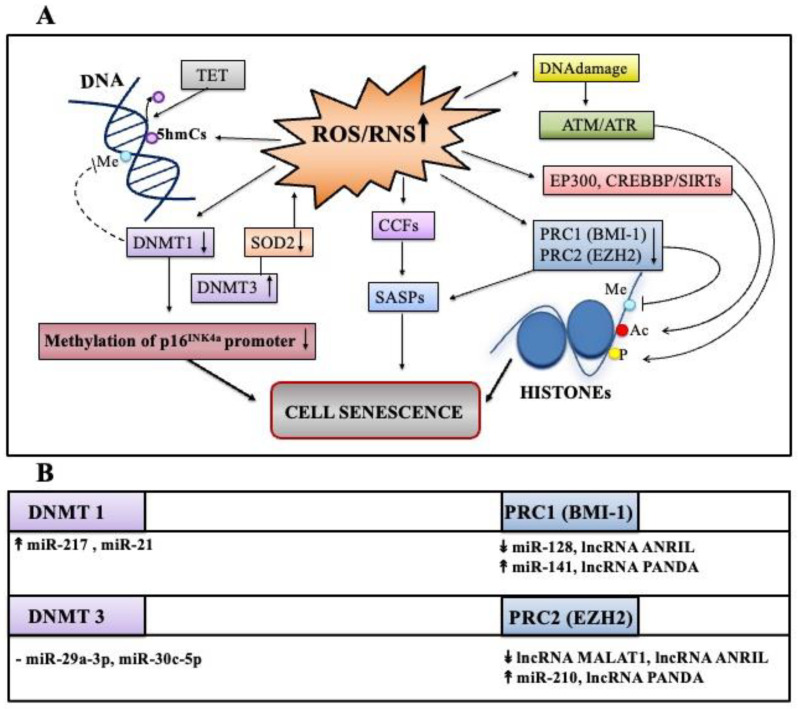
Summary of major epigenetic landscape through ROS/RNS. (**A**) ROS attack cytosines methylated at C5 (5mC), which become 5-hydroxymethylcytosines (5hmCs). These latter can be further oxidized by TET enzymes prompting local DNA demethylation. Increased activity of DNMT3 reduces SOD2 expression that provokes ROS elevation, and consequently, senescence. On the contrary, ROS/RNS reduce the expression of DNMT1 with consequent demethylation of p16^INK4a^ promoter and increased expression of p16. ROS/RNS can directly generate DNA damages, which are engaged by the kinase ATM/ATR that phosphorylates the serine 139 of the histone H2AX variant. ROS/RNS can reshape histone modifications (such as acetylation and methylation) by altering enzymatic reactions of the histone acetylases/deacetylases, such as EP300 or CREBBP/SIRTs and/or histone methylases/demethylases, such as EZH2, BMI-1 of the PRC complexes. Mitochondrial ROS induce the release of cytoplasmic chromatin fragments involved in SASP stimulation. (**B**) Overview of non-coding RNAs deregulated in senescence that can impact epigenetic landscape through ROS/RNS signaling pathways. Up and down arrows indicate ncRNA levels that have been found as increased or decreased, respectively in various models of oxidative stress-induced senescence.

ROS/RNS imbalance can produce an alteration in the epigenetic landscape directly or indirectly and ROS/RNS amounts could be regulated by epigenetic mechanisms [24,152]. Epigenetic modifications include DNA methylation, histone modifications, and non-coding RNA activities, which can also work together to sustain the complex program of senescence [31]. One of the recent examples is a regulatory circuit reported by Jung et al., in which increased DNA methyl transferase 3 (DNMT3) activity due to the decrease of specific microRNA, impacts negatively on mitochondrial SOD2 expression, and consequently, induces senescence through ROS elevation [153] (Figure 4A).

#### 3.3.1. ROS/RNS and DNA Methylation

ROS and RNS can influence the status of DNA methylation. ROS act directly to modify the methylation status, while the RNS mechanism is less elucidated. The hydroxyl radicals attack cytosines normally methylated at C5 (5mC), which become 5-hydroxymethylcytosines (5hmCs). The 5hmCs can be further oxidized by the ten-eleven translocation methylcytosine dioxygenases (TET) enzymes, and subsequently, this provokes local DNA demethylation [154,155]. ROS can also dampen cytosine methylation by influencing the DNMT enzymatic activities involved in methylation of DNA. It has been reported that H_2_O_2_-induced senescence in normal human cells from different origins is accompanied by decreased expression of DNMT1, thus fostering demethylation of p16^INK4a^ promoter region with consequent expression of p16 [156,157]. Finally, differential methylation has been reported in senescent cell genome: It seems that hypermethylation of cell-cycle gene promoters, and hypomethylation of constitutive heterochromatin enhances genome instability, thus further promoting permanent arrest (reviewed in [158,159]).

#### 3.3.2. ROS/RNS and Histone Modifications

As previously discussed, ROS/RNS can directly produce DNA damages that are engaged by the kinase ATM/ATR, which is specifically involved in the most frequent histone post-translational modifications, namely the phosphorylation on serine 139 of the histone H2AX variant, a DDR marker [42,57]. ROS/RNS can also increase or decrease other histone modifications, such as acetylation and methylation by affecting enzymatic reactions catalyzed by the histone acetylases/deacetylases (HATs/HDACs) and by histone methylases/demethylases (HMT/HDM), respectively (Figure 3A).

Histone acetylation/deacetylation processes could be shaped by ROS/RNS at various levels, directly or indirectly [42,155,160]. For example, ROS/RNS can directly influence epigenetic modifiers, such as the acetylase EP300 and its paralog CREBBP, due to oxidation of key cysteine residues or to the fact that they are mechanistically linked to the deregulation of the class III deacetylases, namely of sirtuins [140,160,161]. As previously mentioned, in addition to histones, SIRTs govern the activities of numerous transcription factors, such as FOXOs, p53, and in turn, the redox regulatory circuits at different levels [140,160,161].

Histone methylation/demethylation are dynamic events and coordinate the DNA accessibility for transcription factors. Modifications are principally ensured by two cooperating Polycomb group (PcG) complexes: PRC1 (formed by BMI-1, CBX, HPH, RING1) and PRC2 (composed of EED, SUZ12, EZH2, the methyltransferase) [26,162]. ROS/RNS are implicated in a senescence-driving circuit involving EZH2 downregulation and NOX4 increase through a positive feedback [163]. In line with this, EZH2 and BMI-1, as well as other components of the PcG pathway become downregulated in senescence prompting changes in chromatin landscape, increased p16^INK4a^ expression, and establishment of the SASP [164,165] (Figure 4A). Finally, most types of senescent cells possess peculiar areas of heterochromatin called senescence-associated heterochromatin foci (SAHF), responsible for E2F target genes silencing [49], in parallel with pRb/p16 ^INK4a^ activation [166]. SAHF contain heterochromatin markers, such as lysine 9-trimethylated histone H3 (H3K9me3) associated with heterochromatic protein 1 (HP1), high-mobility group HMGA proteins, and depletion of Histone H1 [58]. However, SAHF, similar to other epigenetic changes are contest dependent [31].

Mitochondrial ROS are specifically involved in the production of cytoplasmic chromatin fragments in senescent cells harboring H2AX variant, H3K9me3, and H3K27me3 markers that could stimulate a signaling involved in SASP production [150].

Finally, ncRNAs expression can be influenced by epigenetic modifications, and in particular, lncRNAs can participate in the formation of a specific chromatin-modifying complex to activate or inhibit transcription (Figure 4B).

## 4. Non-Coding RNAs in Oxidative Stress-Induced Senescence

Non-coding RNAs (ncRNAs) comprise a wide range of endogenous transcripts, such as microRNAs (miRNAs), long RNAs (lncRNAs), and circular RNAs (circRNAs), which act as “regulatory” ncRNAs with diverse functions correlated to the regulation of gene expression. Well established regulatory mechanisms of ncRNAs involve transcription, splicing, translation, and stability of mRNAs, as well as genomic control of chromatin status [33,34].

Discoveries over the last decades have highlighted that ncRNAs are highly responsive to different types of stimuli, and thus they are implicated in numerous cellular responses, including oxidative stress-induced senescence [27,35,36,37,38].

### 4.1. MicroRNAs (miRNAs)

The most extensively studied regulatory ncRNAs are microRNAs (miRNAs), which are supposed to supervise the expression of about 60% of human genes, accounting for the possibility that they are essential regulators of most (if not all) physiological processes of organisms, including differentiation, development, aging, as well as metabolic homeostasis [167,168,169,170,171]. Therefore, their dysregulation is widely recognized as a crucial contributor to major human diseases, including cardiovascular, metabolic, and age-related diseases, as well as cancer [171,172,173].

The miRNAs can directly or indirectly modulate the levels of crucial senescence effectors since they recognize complementary sequences generally localized in the 3′ untranslated regions (3′UTRs) of targeted mRNAs and function with a sequence-specific silencing mechanism [174,175,176]. The miRNAs exhibit conserved mRNA target sequences across mammalian species, and by down-modulating post-transcriptionally the expression of numerous different genes (up to hundred for a single miRNA), they can influence crucial signaling networks implicated in a variety of stress responses, including those implicated in redox homeostasis [177,178,179]. Of note, the cellular redox status could also regulate the biogenesis of miRNAs, and thus suggest another complex interplay between ROS/RNS amounts and miRNA production/levels [180].

Numerous miRNAs by targeting molecular effectors of cell senescence and/or crucial players of antioxidant responses lead to oxidative stress and eventually senescence. The selected examples summarize the current knowledge regarding miRNAs in stress-induced senescence (Table 1).

*miR-106a*. The first observation that miRNAs can be involved in cell senescence induced by H_2_O_2_ comes from the study by Li et al. [181]. The authors demonstrated that, in primary human cells, oxidative stress-induced senescence was associated with miRNA changes with most as downregulated. Further analyses showed that the downregulation of miR-106a, a member of the miR-17 family, is involved in a p53-independent increase of p21, as miR-106a directly targets the p21 3′UTR transcript. However, the downregulation of miR-106a alone was not able to induce senescence.

*miR-182*. Li et al. also correlated the upregulation of miR-182 with the decrease of retinoic acid receptor gamma (RARG), an aging-related gene, which was mediated by miR-182 putative binding site/s on RARG 3′UTR [181]. Moreover, the miR-182 was recently found as induced in primary epithelial cells from the fallopian tube upon H_2_O_2_ exposure and its ectopic expression triggers cell senescence through the p53/p21 pathway [182]. Moreover, the p21 increase was lost in cells harboring p53 mutations, and in this context, miR-182 can rather behave as an “Onco-miR” driving the bypass of senescence and fostering tumorigenesis. This oncogenic role of miR-182 has been demonstrated in other types of cancers [183,184].

*miR-17 family and miR-106–363 family*. Hackl et al. reported [185] that four miRNAs, namely miR-17, miR-19b, miR-20a, and miR-106a, belonging to the miR-17 family and to the paralogous cluster miR-106a-363, were critically expressed at low levels both in cellular and organismal aging models. Very recently, Tai et al. confirmed the downregulation of miR-106a-5p along with miR-20b-5p (also a member of the miR-106a-363 family) in three human multipotent stromal cell lines treated with H_2_O_2_ [186]. Specific genes of the p21/CDK/E2F pathway, one of the signaling implicated in senescence, were found as directly downregulated by these miRNAs in human multipotent stromal cells under oxidative stress conditions [186].

*miR-200 family*. H_2_O_2_ exposure of human umbilical vein endothelial cells (HUVEC), a type of cells prone to senescence, leads to induction of the miR-200 family (miR-200a, miR-200b, miR-200c, miR-429, and miR-141) and among them ectopic expression of miR-200c promotes senescence by direct downregulation of zinc finger E-box binding homeobox 1 (ZEB1), a transcription factor accumulating in proliferative cells [187]. The same miRNA was also found as upregulated in H_2_O_2_-induced senescence of primary human cells [181]. Furthermore, the paper by Carlomosti et al. [188] demonstrated that upon H_2_O_2_ exposure, induction of miR-200c inversely correlates with SIRT1, FOXO1, and eNOS expression, which were validated as its direct targets. In this paper, the regulatory loop among miR-200c and SIRT1/FOXO1/eNOS redox-pathway was demonstrated as a possible contributor to endothelial dysfunctions induced by ROS/RNS deregulation [188] that associate with aging, diabetes, as well as with ischemia and reperfusion processes. It is likely that miR-200 induction is mediated by p53 phosphorylation on Ser33, an H_2_O_2_-induced p53 modification that upregulates the transcription of miR-200 family, at least in liver cell death [189]. Similarly, miR-141, another member of miR-200 family was found as upregulated in replicative and H_2_O_2_-induced senescence [190]. The authors demonstrated that in human diploid fibroblasts, the forced expression of miR-141 blocks proliferation and induces senescence by targeting the 3′UTR of BMI-1 mRNA [190]. BMI-1 is a member of the PcG gene family that maintains an adult stem cell pool by repressing the p16^INK4a^ locus, and thus preventing premature senescence [191].

*miR 146 a/b*. Other miRNAs upregulated in H_2_O_2_-induced senescence of HCA2 fibroblasts are miR-146a and miR-146b members [192]. It was shown that the activity of NF-κB was determinant for the induction of miR-146a/b [193] and that miR-146a/b by downregulating IRAK, act as a negative regulator of pro-inflammatory pathways driven by NF-κB [192] and/or the ETS-related gene (EGR-1/3) and AP-1 transcription factors implicated in SASP production [194]. Moreover, the role of miR-146a increase on the decline of NRF2 during rat liver aging levels has been also reported [195]. Finally, miR-146a/b (together with miR-140 and miR-195) have been found as increased in aged bone MSCs [196]. Curiously, the levels of miR-146a were found as decreased in exosomes derived from H_2_O_2_-induced senescent endothelial MSCs, and thus reduction seems to contribute to the alteration of wound healing process [197], but the related pathway/s need to be investigated.

*miR-206*. The muscle related miR-206 is another miRNA very recently associated with H_2_O_2_-induced senescence in bone marrow-derived MSCs [198]. Oxidative stress-induced senescence in these cells was accompanied by the upregulation of miR-206 targeting alkaline phosphatase 3′UTR mRNA, and miR-206 inhibition counteracts MSC senescence in vitro. Since oxidative stress-induced senescence is a side effect of stem cell potentiality, the same authors successfully showed that miR-206 down-modulation in MSC transplanted models has beneficial effects on cardiac function [198]. Interestingly, miR-206 was found elevated in aged mouse muscle [199], and since NRF2 downregulates its expression, an association of NRF2 decline with miR-206 increase during aging could be speculated.

*miR-144*. Sangokoya et al. reported a functional link between miR-144 and NRF2 levels, since the 3′UTR of NRF2 mRNA can be directly targeted by the miR-144 [200]. It is well accepted that the deregulation of miR-144/NRF2 regulatory axis disturbs the redox balance and accordingly miR-144 was found as upregulated in aged monkey muscle and its expression can be reversed by caloric restriction [201].

*miR-125b*. NRF2 transcriptionally activates the miR-125b expression, which is linked to p53 since it directly binds to p53 mRNA and inhibits p53 expression [202], thus reinforcing the concept that a crosstalk between these two transcription factors is necessary in order to set the outcome of stress [203].

*miR-34a, -335, -93*. Targeting antioxidant effectors mediated by miRNAs may increase ROS/RNS levels, and thus induce premature senescence. This was shown for miR-34a and miR-335, which are increased in aged rat kidney and post-transcriptionally inhibited the expression of mitochondrial SOD2 and TXNRD2 (belonging to the thioredoxins system), respectively. This leads to the increased ROS levels and promotes oxidative stress-induced senescence of young mesangial cells [204]. In aged rat liver, the upregulation of miR-34a together with miR-93 is also implicated in the downmodulation of microsomal glutathione S-transferase 1 (MGST1), an enzyme acting in redox-homeostasis mechanisms [205]. Numerous links between miR-34a increase and cell senescence come from data obtained in different aging systems (aged tissues and cells) [205,206,207,208] and in chronic obstructive pulmonary disease (COPD), a lung condition characterized by increased oxidative stress and accelerated senescence [209]. The obtained data showed that SIRT1/6 are direct targets of miR-34a, thus underlying the key role of SIRTs decline in senescence/aging. On the other hand, p53 is involved in the expression of miR-34 family [210]. Therefore, the decrease in SIRT1 via miR-34 a/b/c enhances p53 activity, and thus promotes a cell senescence program [211]. In addition, miR-34a could directly target NAMPT in hepatocytes, thus reducing NAD^+^ biosynthesis, and consequently, SIRT1 activity [212]. Therefore, these factors may act in concert to promote senescence.

*miR-486–5p*. Kim et al. demonstrated that miR-486–5p induces premature senescence in human adipose-derived stem cells by inhibiting SIRT1, [213], and we also showed that this miRNA was significantly upregulated in stress-induced senescence in primary human fibroblast IMR-90 [214].

*miR-570–3p*. Baker et al. found that miR-570–3p, highly expressed in COPD patients, which are affected by a form of accelerated aging, directly downregulated SIRT1 expression. By contrast, miR-570–3p inhibition restored SIRT1 levels [215]. They also found that H_2_O_2_-induced expression of miR-570–3p in airway epithelial cells was associated with an increase of p21 levels through the p38MAPK/AP-1 signaling [209].

*mir-217*. SIRT1 has been found as a direct target of miR-217 in senescence-prone endothelial cells [216]. The authors showed that miR-217 levels are increased in replicative aged cells and that overexpression of miR-217 enhanced FOXO1 acetylation, thus reducing eNOS protein amounts. This could possibly influence the intracellular redox status. Recently, miR-217 has been causally implicated in DNMT1 decrease observed in passage-aged human skin fibroblasts [217]. Finally, miR-217 and miR-21 (discussed below) have been both found as increased in extracellular vesicles derived from replicative senescent HUVECs [218].

*miR-29a/b/c*. The family of miR-29 (miR-29a/b/c) has been associated with the increase of oxidative stress constitutively found during ageing. In fact, members of miR-29 family were found as elevated in different aged tissues, including the heart [219], muscle, and liver [220], as well as brain [221]. Heid et al. found that the high H_2_O_2_ induces expression of miR-29 family in cardiac cells and this modulation counteracted fibrosis in the zebrafish model carrying the miR-29 family knockout [219], whilst Ugalde et al. reported that normal murine fibroblasts did not increase miR-29a/b/c levels upon transient mild H_2_O_2_ exposure, but their upregulation was rather mediated by persistent DNA-damage involving the p53 pathway [222]. The miR-29 family by downregulating the expression of numerous mRNA, such as collagen genes or epigenetic regulators, alleviates the age-dependent fibrosis process [219]. Moreover, a recent work by Ripa et al. associates the increase of miR-29 with age-related iron imbalance demonstrating that miR-29 directly targets iron responsive protein 2 (IRP2), a crucial component of the iron homeostasis [223]. Finally, miR-29 has been linked to premature senescence of hMSCs, where it contributes to epigenetic changes [153]. Early passage hMSCs silenced for DiGeorge critical region 8 (DGCR8), a RNA/heme-binding protein involved in miRNA biogenesis, undergo premature senescence. This was associated with ROS increase, loss of mitochondrial homeostasis, and downregulation of SOD2 transcription. Since in this setting, DNA methyltransferase 3 alpha (DNMT3A) was implicated in senescence-decline, in search for miRNAs targeting 3′UTR of DNMT3A, the authors demonstrated that miR-29a-3p along with miR-30c-5p directly repressed DNMT3A expression. Accordingly, ectopic expression of these miRNAs reduced ROS, restored SOD2 protein levels, and improved mitochondrial functions [153].

*miR-155*. Downregulation of miR-155 has been correlated with oxidative stress-induced senescence [214,224]. The decrease of miR-155 in WI-38 senescent cells causes increased levels of TP53INP1, involved in the p53-mediated growth arrest pathway [224]. The miR-155 is a multifunctional miRNA and can target many transcripts in different cellular contexts, including FOXO3a and HIF-1α, thus contributing to a control of ROS levels. A recent work by Onodera et al. focused on the role of miR-155 during senescence of MSCs [225]. This study demonstrated that miR-155 increased in the bone marrow of aged mice and is involved in ROS generation by suppressing the transcription factor C/EBPβ, which is involved in the regulation of NRF2, SOD1, and HMOX1 expression both in mouse and human MSCs. Since during MSC transplantation, ROS accumulation could limit the efficacy of therapy [226], the same authors demonstrated that MSCs knockout for miR-155 display reduced ROS levels after transplantation [225].

*miR-494*. The miRNAs can contribute to mitochondrial homeostasis, and in addition to cytosol, a small number also localized in the mitochondria (rewieved in [227]). A well-established role of nuclear miRNAs acting on mitochondrial functions (anterograde signals) is provided by miR-494. By targeting mitochondrial transcription factor A (TFAM) and FOXJ3 mRNAs, miR-494 regulates mitochondrial biogenesis [228]. Other miR-494 targets that can contribute to mitochondrial homeostasis in different cell contexts, include SIRT1, c-Myc [229], DJ/PARK7, a redox-related chaperon [230], and PGC-1α [231]. In addition, some data indicate a causal role of miR-494 in cell senescence, which is induced by oxidative stress or telomere shortening [214], as well as by DDR-inducing agents [232]. We demonstrated that overexpression of miR-494 in normal IMR-90 fibroblasts induced premature senescence and increased ROS, which can in turn, feedback positively to DDR, thereby sustaining the senescent program [214]. Further analyses revealed that miR-494 directly targets hnRNPA3 and RAD23B 3′UTRs, whose reduced expression was causally related to senescence [233].

*miR-128*. One of the first miRNAs to be implicated in oxidative stress-induced senescence was miR-128 that directly targets BMI-1 3′UTR in medulloblastoma cells [234]. Bone marrow and thymocyte cells deficient in BMI-1 displayed increased ROS levels along with mitochondrial dysfunctions. These conditions sustain and engage a DDR signal mediated by CHK2/RAD53, independently of p16^INK4a^ pathway activation [235]. Moreover, miR-128 controls the redox-state also through direct regulation of multiple downstream transcripts, including SIRT1 [236], MAFG, a positive interactor of NRF2 [237], and thioredoxin interacting protein (TXNIP) [238].

*miR-15b*. Lang et al. recently demonstrated that miR-15b directly targets the mitochondrial deacetylase SIRT4 3′UTR. The authors found that SIRT4 increased in stress-induced and replicative senescence, as well as in photoaged human skin in vivo. In addition, miR-15b downregulation inversely correlated with SIRT4 protein levels in various senescence models. Accordingly, the enforced inhibition of miR-15b increased SIRT4 expression, which was associated with mitochondrial ROS production that fosters mitochondrial dysfunctions, a hallmark of aged cells [239].

*miR-210*. Mitochondrial functions can be also modulated by miR-210. Specifically, miR-210 increases ROS production via the inhibition of the electron transport chain (ETC) components, such as iron-sulfur cluster scaffold homolog (ISCU) and cytochrome-c oxidase assembly protein (COX10) [240], as well as by targeting some subunits of the complexes I and II [241]. In fact, ectopic expression of miR-210 induced premature cell senescence in human lung IMR-90 fibroblasts [214]. In searching for miRNAs linked to p16-mediated senescence of human normal mammary epithelial cells, Overhoff et al. found that miR-210 directly targets the 3′UTRs of components of the PcG pathway, namely EED, EZH2, and SUZ12, that consequently mediated the epigenetic increase of p16 [242].

*miR-663*. Premature senescence induced by H_2_O_2_ in normal WI-38 cells induced miR-663 levels (together with miR-34a and miR-638) [243]. The functions of miR-663 are not well defined in stress-induced senescence. This miRNA is pleiotropic, and its dysregulation correlates with different types of stresses, depending on the genetic contexts and/or the stressor types [244,245]. Recently, miR-663 has been also associated with mitochondria-to-nucleus retrograde communication [246]. Unusually, by direct targeting the 3′UTR of ubiquinol-cytochrome C reductase complex assembly factor 2 (UQCC2), miR-663 stabilizes the UQCC2 transcripts, thus improving the function of ETC complexes in cancer progression [246].

*miR-21*. Very recently, Mensà et al. reported that miR-21 along with miR-217 is the most enriched miRNA in extracellular vesicles derived from senescent endothelial cells and that both miRNAs directly target DNMT1 [218]. Extracellular vesicles enriched in miR-21 can be transferred to the surrounding young cells, where it can spread signals for an epigenetic program inducing senescence through modulation of DNMT1/SIRT1 levels [218]. A considerable number of studies indicate that mir-21 has a pro-fibrotic and pro-inflammatory role in different models (reviewed in [247]). Since miR-21 could directly target antioxidant genes (i.e., SOD2) or indirectly activate ROS producing enzymes (i.e., NOX4), it can be speculated that the increase in miR-21 functionally contributes to oxidative stress-induced senescence, an important factor in fibrosis. Remarkably, anti-miR-21 delivery improves the cardiac function in animal models of heart failure, decreasing cardiac fibrosis and hypertrophy [248]. According to this, miR-21 has been proposed as a candidate biomarker of healthy aging since decreased miR-21 levels have been found in healthy people older than 80 years and centenarians, whereas it was increased during aging and age-related conditions, arguing that lower levels could be beneficial for longevity [249].

*miR-22*. During in vivo heart aging, cardiac fibroblasts displayed elevated expression of various pro-senescent miRNAs, including miR-22 [250]. In heart fibroblasts, miR-22 selectively targeted the 3′UTR of mimecan/osteglycin mRNA involved in collagen fibrillogenesis, whilst in epithelial cells it downregulated SIRT1/CDK6 levels [250]. Very recently, the miR-22 increase has been associated with mitochondrial ROS induction during ionizing radiations in rat bone marrow mesenchymal stromal cells. The authors found that miR-22 directly targets the 3′UTR of the redox-related Redd1/DDIT4 transcript [251].

**Table 1:** List of microRNAs whose expression and targets/pathways are implicated in oxidative stress-induced senescence. Luciferase assay not reported.

MicroRNA	Expression Pattern	Target/Pathway	Cell Models/ Diseases	References
**miR-106a**	down	p21	HDFs; HDFs (HTMs)	[181]
**miR-182**	up	RARG	HDFs; HDFs (HTMs)	[181]
	up	p53/p21 pathway	FTEs	[182]
**miR-17 family and** **miR-106a-363 family**	down	-	HDFs; HUVECs; RPTECs; T cells (CD28+); Isolated BMSCs, FSK-MSCs, T cells (CD28+)	[185]
	down	p21/CDK/E2F pathway	WJ-MSCs	[186]
**miR-200c**	up	ZEB1	HUVECs, MEFs	[187]
	up	-	HDFs, HDFs (HTMs)	[181]
	up	SIRT1, FOXO1, eNOS	HUVECs	[188]
**miR-141**	up	BM1-1/p16^INK4a^ pathway	HDFs (MRC5)	[190]
**miR-146a/b**	up	IRAK1 (?), NF-κB pathway	HDFs (HCA2, BJ, IMR-90)	[192]
	up	TRAF6/IRAK1/2, HuR, NF-κb, EGR-1/3, AP-1 pathway	HUVECs	[194]
	up	NRF2	Rat hepatocytes	[195]
	up	-	Isolated BMSCs	[196]
	down in exosomes	-	Endothelial MSCs	[197]
**miR-195**	up	Tert	Isolated BMSCs	[196]
**miR-206**	up	Alpl	BMSCs	[198]
	-	-	Aged mouse muscle	[199]
**miR-144**	up	NRF2	Aged rhesus monkey’s muscle	[201]
**miR-125b**	-	p53	SH-SY5Y	[202]
**miR-335**	up	MnSOD	Aged rat kidney tissues, mesangial cells	[204]
**miR-93**	up	MGST1, SIRT1	Aged rat liver	[205]
**miR-34a**	up	TXNRD2	Aged rat/mouse tissues, mesangial cells	[204]
	up	MGST1	Aged rat liver	[205]
	up	-	HUVECs, HAECs, HMVECs	[206,208]
	up	SIRT1/6	BEAS2Bs; COPD	[209]
	up	NAMPT	Isolated hepatocytes	[212]
**miR-486-5p**	up	SIRT1	hAT-MSCs	[213]
**miR-570-3p**	up	SIRT1, p21; p38MAPK/AP-1 signaling	BEASs, SAECs; COPD	[215]
**miR-217**	up	SIRT1; SIRT1/FOXO1 pathway	HUVECs, HAECs, HCAECs	[216]
	up	DNMT1	Isolated HSFs	[217]
	up	-	HUVECs	[218]
**miR-29a/b/c**	up	Col1a1 col1a2, col15a1 DNMT3A, DNMT3B	Aged tissues (heart, muscle, liver, brain), premature aging model (liver)	[219,220,221,222]
	up	IRP2	Isolated murine neurons, vertebrate aged models	[223]
	-	DNMT3A	hAT-MSCs	[153]
**miR-30c-5p**	-	DNMT3A	hAT-MSCs	[153]
**miR-155**	down	-	HDFs (IMR-90)	[214]
	down	TP53INP1(?); p53 pathway	HDFs (WI-38)	[224]
	up	C/EBPβ	Aged mouse bone marrow tissues	[225]
**miR-494**	up	hnRPA3, RAD23B	HDFs (IMR-90)	[233]
**miR-128**	down	BM1-1/p16^INK4a^ pathway	Daoy/D283, tissues from BMI-/- mice	[234]
**miR-15b**	up	SIRT4	HDFs (F623, F357), photoaged human skin samples	[239]
**miR-210**	up	-	HDFs (IMR-90)	[214]
	up	EED, EZH2, SUZ12	HMECs	[242]
**miR-663**	up	-	HDFs (WI-38)	[243]
**miR-21**	up	DNMT1/SIRT1	HUVECs, HAECs, HCAECs	[218]
**miR-22**	up	Mimecan/osteoglycin, SIRT1, CDK6	Aged mouse heart, neonatal HCFs	[250]
	up	Redd1/DDIT4	rBMSCs	[251]

### 4.2. Long RNAs (LncRNAs)

Long non-coding RNAs (LncRNAs) include multiple RNA species longer than 200 nucleotides, which are not translated into proteins. They are now considered crucial regulators in the gene expression acting both in nuclear and cytoplasmic compartments, and at different levels.

They control the architecture and remodeling of chromatin, the transcriptional process by influencing RNA polymerase II activities and stability, translation, as well as post-translational modifications of transcripts (reviewed in [252]). Therefore, lncRNAs show a plethora and diversity of functions. As a result, studies aimed at understanding their roles are rapidly increasing. The altered expression of several lncRNAs has been associated with oxidative stress-related conditions, including senescence. However, it is important to note that, among the several hundred lncRNAs currently known, only for a few of them the specific functions in oxidative stress response/s have been deeply studied [27,37,253,254,255,256,257] (Table 2).

Abdelmohsen et al. performed a comprehensive evaluation of lncRNAs differentially expressed in senescent human diploid WI-38 fibroblasts by RNA sequencing (RNA-Seq) [257]. They found a number of antisense transcripts, pseudogene-encoded, and previously annotated lncRNAs, which display an altered expression in senescence (up- or downregulated). To our knowledge, this was the first study reporting a collection of senescence-associated lncRNAs.

*lncMALAT1*. Abdelmohsen et al. demonstrated that the downregulation of MALAT1 is causally involved in senescence [257]. Recently, MALAT1 has been linked to the protective NRF2 signaling in HUVECs during oxidative stress induced by H_2_O_2_. In this context, the forced expression of MALAT1 increases resistance to stress, while its knockdown has the opposite effect. At molecular levels, MALAT1 overexpression reduced KEAP1 protein, thus favoring nuclear NRF2 accumulation [258]. This could be mediated by the methyltransferase EZH2, which affects the histone methylation status inhibiting KEAP1 transcription [259]. Other possible links of MALAT1 with senescence and/or oxidative-degenerative conditions could be mediated by the NF-κB/iNOS pathway [260] and/or p38MAPK signaling cascade [261], but this has not been demonstrated yet in senescence.

*lncMIAT*. Another lncRNA, found downregulated in human diploid senescent WI-38 fibroblasts by Abdelmohsen et al., was MIAT [257]. Recently, Zhao et al. verified that lncMIAT is also reduced and causally involved in H_2_O_2_-induced cell senescence in hepatocellular carcinoma [262]. LncRNAs may act as competitive endogenous RNAs (ceRNAs) to sponge miRNAs, and thus release the transcripts targeted by the specific miRNA/s from translational inhibition and/or degradation [263]. By the intersecting available databases from gain and loss-of-function experiments, the same authors indeed found that MIAT acts as a ceRNA for miR-22–3p, which directly targets SIRT1 3′UTR. Therefore, knockdown of MIAT, by releasing miR-22–3p, decreased SIRT1 levels and promoted senescence through p53/p21 and p16/pRb pathways [262].

*lncH19*. In another model of H_2_O_2_-induced senescence using central nucleus pulposus cells (NPCs), miR-22 was also found to be sponged by lncH19, a multifaceted lncRNA which is increased in various degenerative conditions [264]. The authors demonstrated that H19 is causally implicated in the effects of H_2_O_2_ and that the increased H19 by sequestering miR-22, a direct inhibitor of the lymphoid enhancing factor-1 (LEF1), could activate the downstream Wnt/β-catenin signaling contributing to degenerative events [264]. Similarly, the high H19 has been recently implicated in cardiomyocyte senescence functioning as a ceRNA for miR-19a [265]. In this work, Zhuang et al. demonstrate that the increase of H19, by scavenging miR-19a, consequently upregulated the suppressor of cytokine signaling 1 (SOCS1) expression (a target of this miRNA), thus enhancing the p53/p21 pathway [265]. Recently, H19 was found as downregulated in vascular endothelial senescence and its decline in vivo reduces angiogenesis and favors a pro-inflammatory environment associated with IL-6 signaling and STAT3 pathway activation [266]. Finally, other studies exploiting a miRNA crosslinking and immunoprecipitation (miR-CLIP) appoach, previously identified that miRNA partners of H19 comprise members of the miR-17r–5p family [267]. However, the implication of lncH19/miR-17–5p axis in stress-induced senescence has not been fully elucidated yet.

*lncANRIL*. The decrease of the antisense noncoding RNA of the INK4 locus, ANRIL, has been often associated with senescence. In fact, ANRIL can directly recruit the polycomb repressive complexes PRC1 and PRC2 to repress the neighbor INK4b-ARF-INK4a locus encoding p15INK4b, p14ARF, and p16^INK4a^, respectively. In this case, ANRIL inhibits senescence sustaining proliferation (reviewed in [268]). Very recently, Tan et al. reported that ANRIL antagonized the senescence of vascular smooth muscle cells by sponging miR-181a, which directly targets 3′UTR SIRT1 mRNA, thus reducing the cell cycle arrest mediated by the p53/p21 pathway [269]. Moreover, Du et al. demonstrated that ANRIL overexpression alleviated H_2_O_2_-induced cell injury in human lens epithelial cells by mediating miR-21 upregulation that activates the antioxidant AMPK/β-catenin pathway. Importantly, ANRIL and miR-21 exhibited a decreased expression in cataract patient tissues [270].

*lncHOTAIR*. The lncHOTAIR is directly upregulated by NF-κB in response to DNA damage, and its possible contribution to the positive-feedback loop cascade during DDR-induced signal senescence has been reported in ovarian cancer [271]. Yoon et al. first demonstrated that levels of HOTAIR increase during senescence, and that in this case, it acts as a platform to recruit selected proteins for the ubiquitin–proteasome pathway [272]. Recently, HOTAIR has been implicated in oxidative stress-induced damages in cardiomyocytes [273]. In H9c2 cells, HOTAIR targets miR-125, and knockdown of HOTAIR significantly increased miR-125 levels in both normal and oxidative stress conditions. As matrix metalloproteinases-2 (MMP2) is a direct target of miR-125, the HOTAIR/miR-125/MMP2 signaling pathway has been proposed to regulate the oxidative stress response upon myocardium injury [273]. In addition, HOTAIR is increased in degenerative conditions of human NPCs driving premature senescence, apoptosis, and ECM degradation [274].

*lncPANDA*. Levels of lncRNA PANDA are regulated by p53 and were increased in replicative and oncogenic RAS-induced senescent cells [275]. At molecular levels, in proliferating cells, PANDA interacts with hnRNPU (also called scaffold-attachment-factor A (SAFA)), a scaffold protein that binds ncRNAs and PRC1/2 components, thus recruiting a repressive complex on the genes that activate senescence, such as p21. During senescence, since SAFA is decreased, PANDA sequesters the NF-YA transcription factor, thus impeding the expression of NF-YA-dependent proliferative genes. According to this, PANDA depletion in BJ fibroblasts promotes senescence entry [275]. However, the PANDA implication in stress-induced senescence has not been investigated yet.

*lincRNA-p21*. Recently, Xia et al. reported that long intergenic (linc) RNA-p21 was upregulated in bone marrow MSCs isolated from aged mice, and this is associated with increased intracellular ROS production and reduced Wnt/β-catenin pathway [276], a signal cascade declining during stem cell ageing [277]. The authors found that knockdown of lincRNA-p21 increased proliferation of aged MSCs, decreased ROS generation, and these effects were abolished by silencing β-catenin, thereby linking lincRNA-p21, oxidative stress, and Wnt/β-catenin pathway [276]. LincRNA-p21 is a transcriptional target of p53 and cooperates with hnRNPK to the p53-mediated activation of p21 transcription, albeit this activity was unrelated to the senescence program [278].

*lncUCA1*. Kumar et al. showed that during senescence lncRNA UCA1 sequesters hnRNPA1, a RNA-binding protein destabilizing p16^INK4a^ transcripts, thus contributing to improve p16^INK4a^ protein levels [279]. In fact, UCA1 transcript levels were increased in premature senescence induced by oncogenic RAS and accordingly its forced expression rapidly induced senescence. The same authors also reported that in young cells, UCA1 transcription is directly inhibited by a repressor complex composed of a coactivator of AP1 and estrogen receptor/T-box transcription factor3 (CAPERα/TBX3), also involved in p16^INK4a^ and Rb silencing [279].

*vlincRNA VAD*. The very long intergenic non-coding (vlinc) RNA antisense to DDAH1 (VAD) also modulates the chromatin structure of the p16^INK4a^ locus. Lazorthes et al. first demonstrated that the transcription of VAD was strongly induced during oncogenic RAF-induced senescence and that ectopic silencing of VAD in senescent cells reversed some features of senescent phenotype [280]. At molecular levels, VAD contributes to the transcriptional activation of INK4a locus by blocking the incorporation of the repressive histone variant H2A.Z at the promoter of INK4a gene [280]. Since DDAH1 is related to the NO signaling [281], an important pathway in cardiovascular physiology, a functional link between VAD and oxidative stress-related senescence could be speculated, albeit not investigated in that paper.

**Table 2:** List of lncRNAs whose expression and targets/pathways are implicated in oxidative stress-induced senescence.

LncRNA	Expression Pattern	Target/Pathway	Cell Models/ Diseases	Reference
**MALAT1**	down	-	HDFs (WI-38)	[257]
	down	Keap1/NRF2 pathway	HUVECs	[258]
	down	EZH2	MMs	[259]
	down	NF-κB/iNOS pathway	Endometrial tissues	[260]
	down	p38/MAPK signaling	HLECs	[261]
**MIAT**	down down	- miR-22-3p	HDFs (WI-38) Hepatocellular carcinoma tissues, HCC	[257] [262]
**H19**	up up down	miR-22/Wnt/β catenin miR-19a IL-6 signaling/ STAT3 pathway	NCPs Isolated NMVCs Aged mice tissues, HUVECs	[264] [265] [266]
**ANRIL**	down down	miR-181/SIRT1 miR-21	VSMCs Patient tissues, HLECs	[269] [270]
**HOTAIR**	up up up	- Protein ubiquitination miR-125	Epithelial ovarian cancer cells, ovarian tissues HeLa, HDFs (IDH4, WI-38) H9c2S	[271] [272] [273]
**PANDA**	up	SAFA/hnRNPU; NF-YA	HDFs (BJ, WI-38)	[275]
**LincRNA-p21**	up	Wnt/β catenin	Isolated BMSCs	[276]
**UCA1**	up	hnRNPA1	HDFs (HFFs), MEFs	[279]
**VAD**	up	INK4 locus	HDFs (WI38- hTERT RAF1-ER, IMR90)	[280]

### 4.3. Circular RNAs (circRNAs)

The role of circRNAs in oxidative stress-induced senescence is underexplored. The circRNAs are a type of endogenous non-coding RNA molecules that are covalently close and mainly produced by a non-canonical splicing event called “back-splicing” (reviewed in [282,283,284]). The roles of circRNAs in different physiological and pathological processes, including cardiovascular diseases and neurological disorders, as well as cancer have been reported [283,284]. Most of these circRNAs act by sequestering miRNAs (miRNA sponges/decoys), while others can interact with RNA-binding proteins to suppress or enhance their activities. In addition, they can function as scaffolds for complex assembly and localization [284] (Table 3).

*circPVT1*. A group of circRNAs deregulated (up- or downregulated) in replicative senescence of WI-38 cells were recently identified by Panda et al. [285]. Among the downregulated circRNAs, circPVT1 was causally related to senescence since its silencing favors the induction of senescence specific markers, including the increased level of p53 protein. The authors through pull-down experiments also demonstrated that circPVT1 acts as a sponge for let-7, a miRNA involved in senescence [243]. Therefore, cirPVT1, by inhibiting let-7 activity, elevated cell proliferation and this was mediated by IGF2BP1, KRAS, and HMGA2 protein increases, whose transcripts are direct targets of let-7 [285].

*circFOXO3*. Levels of circFOXO3 are induced in aged murine tissues (hearts, lung, derma, and intestine), as well as in different cell lines upon H_2_O_2_ treatments. The circFOXO3 has been implicated in cardiac senescence since its silencing reduces senescence markers in vitro and attenuated doxorubicin-induced cardiomyopathy in mice. In search for possible interactors of circFOXO3, the authors examined its effects on ID, HIF1α, E2F1, and FAK protein localization and demonstrated that circFOXO3 interacted and trapped these factors in the cytoplasm upon H_2_O_2_ treatments, thus inhibiting their functions [286].

*circCCNB1*. Yu et al. found circCCNB1 strongly under-expressed in premature senescence induced by irradiation of 2BS fibroblasts [287]. Ectopic reduction of circCCNB1 in 2BS cells induced the expression of p53, p21, and p16 proteins. The authors also demonstrated that circCCNB1 directly sponges miR-449a, thus preventing its binding to the 3′UTR of CCNE2 (Cyclin E2) mRNA, and thus inhibiting the senescence process [287].

*circGNAQ*. Very recently, Wu et al. correlated circGNAQ to the senescence of endothelial cells [288]. The levels of circGNAQ were reduced in senescent endothelial cells, as well as in aorta tissues from aged mice and in human blood samples from old people. The authors reported that silencing of circGNAQ accelerated senescence and induced p16 protein levels accompanied by reduced angiogenesis, while the overexpression has opposite effects. Moreover, by in vivo precipitation of circRNAs, the authors identified a number of candidate miRNAs. Among these miRNAs, they found the binding with miR-146a–5p, which was particularly increased. Further analyses demonstrated that circGNAQ sponges miR-146a–5p, which functionally affects the expression of polo-like kinase2 (PLK2), an enzyme implicated in cell cycle progression. Of interest, mice injected with circGNAQ showed reduced signs of atherosclerosis after the high fat diet [288].

*circERCC2*. Xie et al. extracted nine downregulated circRNAs from human publicly expression datasets of intervertebral disc disease (IVDD) and confirmed that circERCC2 was the most decreased in IVDD samples [289]. Similarly, bioinformatic analyses on miRNA datasets (further confirmed in IVDD samples) bring out miR-182–5p as a putative target of circERCC2. Moreover, the ectopic expression of circERCC2 in NPCs inhibited deleterious effects of the oxidative stress inducing agent tert-Butyl hydroperoxide on mitochondria (TBHP). Similar results were obtained upon miR-182–5p silencing. These effects are mediated by SIRT1, a downstream target of miR-182–5p [289].

## 5. Conclusions

Even though cell senescence is a physiological program to arrest proliferation of damaged/harmful cells, this process is now considered an important contributor in aging and age-related pathologies. Chronic increase of oxidative stress is a driving factor in the development of cell senescence and consequent age-related functional decline. Accumulation of senescent cells impacts on the functionality of organs/tissues and senescence of adult stem cell pools also hinders tissue renewal in vivo. Therefore, drugs/treatments or interventions that can specifically contribute to fine-tune the redox cellular status are considered possible strategies to treat or even prevent the accumulation of senescent cells, and thus age-related diseases. Senotherapeutic and senomorphic drugs that specifically target senescent cells are considered promising strategies, but limited by the lack of studies on long-term effects in humans.

Epigenetic changes that were previously considered imprinted for a specific gene expression profile, nowadays are indeed considered a dynamic event as transcription itself. Epigenetic therapies based on ncRNAs are promising, but still very much in infancy. However, mild perspectives on clinical usages of miRNAs are now emerging as candidate diagnostic circulating biomarkers. Future studies on specific in vivo functions of lncRNAs are needed to shed light on their impacts on oxidative stress-induced senescence since researchers have only recently started to ascribe precise functions for lncRNAs. Moreover, studies on circRNAs as new players in redox regulatory networks, as well as in cell senescence are a very open field. Systematic assessment by gain and loss-of-function and/or by circRNA-miRNA-RBP/s (RNA binding protein/s) interaction studies will be important to better define circRNA role/s in senescence context, and to achieve the results appropriate to address research for therapeutic goals.

## Figures and Tables

**Table 3:** List of circRNAs whose expression and targets/pathways are implicated in oxidative stress-induced senescence.

circRNA	Expression Pattern	Target/Pathway	Cell Models/ Diseases	Reference
**circPVT1**	down	Let-7/IGF2BP1, KRAS, HMGA2	HDFs (WI-38)	[285]
**circFOXO3**	up	ID1, E2F1, HIF1α, FAK	Aged human and murine heart tissues, Isolated cardiomyocytes, MEFs, MCFs, NIH3T3, B16	[286]
**circCCNB1**	down	miR-449a/CCNE2	HDFs (2BSs, IMR-90)	[287]
**circGNAQ**	down	miR-146a-5p/PLK2	Aged aorta tissues, blood from old peoples HUVECs, HCAECs	[288]
**circERCC2**	down	miR-182-5p/SIRT1	NPCs, IVDD	[289]

## Abbreviations

2BS	human fetal lung diploid fibroblasts
AKT/PKB	serine/threonine-protein kinase 1/protein kinase B
BEAS-2B	human non-tumorigenic lung epithelial cells
BJ	human normal foreskin fibroblasts
BMI-1	B cell-specific Moloney murine leukemia virus integration site 1
BMSCs	bone marrow mesenchymal stem cells
C/EBP	CCAAT/enhancer-binding protein beta
COPD	chronic obstructive pulmonary disease
D283	medulloblastoma cell line
Daoy	medulloblastoma cell line
DNMT 1	DNA methyl transferase 1
DNMT3	DNA methyl transferase 3
EZH2	enhancer of zeste 2 polycomb repressive complex 2 subunit
FOX	Forkhead box
FSK-MSCs	human foreskin-mesenchymal stem cells
FTEs	fallopian tube epithelial cells
GATA4	GATA binding protein 4
H9c2s	rat cardiomyoblast cells
HAECs	human aortic endothelial cells
hAT-MSCs	human adipose tissue-derived mesenchymal stem cells
HATs	histone acetylases
HCA2s	human foreskin fibroblasts
HCAECs	human coronary artery endothelial cells
HCCs	hepatocellular carcinoma cell lines
HCFs	human cardiac fibroblasts
HDACs	histone deacetylases
HDFs (F623, F357)	primary human dermal fibroblasts
HDFs	human diploid fibroblasts
HDM	histone demethylases
HFFs	human foreskin fibroblasts
HLECs	human lens epithelial cells
HMECs	human normal mammary epithelial cells
HMT	histone methylases
HMVECs	microvascular endothelial cells
HSFs	human skin fibroblasts
HTMs	human trabecular meshwork cells
HUVECs	human umbilical vein endothelial cells
IDH4	1MR90-D305.2H4 cell line
IMR-90	human fetal lung fibroblasts
IVDD	intervertebral disc disease
JNK	c-Jun N-terminal kinase
MCFs	mouse cardiac fibroblasts
MEFs	mouse embryonic fibroblasts
MMs	multiple myeloma cell lines
SOD1	copper and zinc superoxide dismutase
SOD2	manganese superoxide dismutase
MRC5s	human fetal lung fibroblasts
NAD^+^	nicotinamide adenine dinucleotide
NAMPT	nicotinamide phosphoribosyltransferase
NF-κB	nuclear factor kappa-light-chain-enhancer of activated B cells
NIH3T3	embryonic fibroblast cell line
NMVCs	neonatal mouse ventricular cells
NPCs	nucleus pulpous cells
NRF2	nuclear factor erythroid 2-related factor 2
PARP1	poly-ADP ribose polymerase 1
PcG	polycomb group
PGC-1α	PPARγcoactivator 1 alpha
PRC1	polycomb repressive complex 1
PRC2	polycomb repressive complex 2
rBMSCs	rat bone marrow mesenchymal stromal cells
RPTECs	primary renal proximal tubule epithelial cells
SAECs	human primary small airway epithelial cells
SAHF	senescence-associated heterochromatin foci
SASP	senescence-associated secretory phenotype
SH-SY5Y	human neuroblastoma cell line
SIRTs	sirtuins
TET	ten-eleven translocation methylcytosine dioxygenases
VSMCs	vascular smooth muscle cells
WI-38	human fetal lung fibroblasts
WI38- hTERT RAF1-ER	immortalized human fetal lung fibroblast cells
WJ-MSCs	Wharton’s jelly-derived mesenchymal stromal cells
